# Oral health and infective endocarditis among high-risk individuals: a nationwide register-based study

**DOI:** 10.1080/20002297.2026.2684117

**Published:** 2026-06-12

**Authors:** Niko Vähäsarja, Bodil Lund

**Affiliations:** a Department of Dental Medicine, Division of Oral Diagnostics and Rehabilitation, Karolinska Institutet, Stockholm, Sweden; b Medical Unit of Plastic Surgery and Oral and Maxillofacial Surgery, Karolinska University Hospital, Stockholm, Sweden

**Keywords:** Infective endocarditis, oral health, periodontitis, caries, recurrence, secondary prevention

## Abstract

**Background:**

Poor oral health has long been hypothesized as a contributor to infective endocarditis caused by *Viridans* group streptococci (VGS-IE), particularly among individuals at increased risk of the disease. However, large-scale population-based evidence remains sparse.

**Objective:**

Assess the association between severe oral disease and IE among high-risk individuals using Swedish nationwide health registers.

**Design:**

Nested case-control study conducted within a national cohort of 64,634 individuals at high risk of IE due to prior IE, prosthetic heart valves, or congenital heart disease (CHD), followed between July 1, 2008, and December 31, 2018. 881 cases of IE and 8,810 controls were identified using the National Patient Register and the Swedish Registry of Infective Endocarditis. Poor oral health was defined using the Swedish Dental Health Register. Conditional logistic regression was used to estimate odds ratios (ORs) for poor oral health in cases versus controls, adjusted for age, sex, educational attainment and comorbidities.

**Results:**

Among individuals with prior IE, poor oral health was associated with both all-cause IE and VGS-IE. All-cause IE: adjusted OR 3.02 (95% CI 1.95–4.69). VGS-IE: adjusted OR 2.23 (95% CI 1.02–4.90). Among those with prosthetic valves, no significant associations were observed (all-cause IE: adjusted OR 1.47 [95% CI 0.94–2.30]; VGS-IE: adjusted OR 1.09 [95% CI 0.44–2.71]). No conclusions could be drawn for the CHD-group due to a lack of data.

**Conclusions:**

Poor oral health was associated with IE recurrence among individuals with a history of IE, suggesting a role for oral health in prevention strategies.

## Introduction

Infective endocarditis (IE) remains a life-threatening condition with an in-hospital mortality rate of 5–20%, despite advances in antimicrobial therapy and cardiac surgery [1,2]. A key mechanism is microoganisms forming a microbial colonisation on heart valves. *Viridans* group streptococci (VGS), common constituents of the oral microbiota, cause some 25–30% of cases [3–5]. Historically, the connection between dental health and VGS-IE was underscored by recommendations for antibiotic prophylaxis (AP) prior to invasive dental procedures (IDPs), especially in individuals at high risk for IE. However, after the 2007 American Heart Association and subsequent European guideline revisions, many countries narrowed or discontinued AP in dentistry due to a lack of conclusive evidence of its benefit [6,7]. Notably, Sweden ceased routine recommendations for dental AP for high-risk individuals in October 2012.

High-risk individuals, defined by a history of IE, presence of a prosthetic heart valve, or complex congenital heart disease (CHD) have incidence rates of IE 100-fold higher than the general population [1,3,8–10]. Despite this elevated baseline risk, prior studies from Sweden found no significant increase in IE following the 2012 AP policy change [11]. These observations align with analyses from England, where no clear spike in streptococcal IE was detected after AP was halted in 2008 [4].

Several register based studies focus on the association between IDPs and IE among high risk individuals [12,13]. It is uncertain if the associations found are due to dental treatment, or poor oral health resulting in IDPs.

In Sweden, a previous study found no association between IDPs and IE among 76,762 high risk individuals, however, the frequency of poor oral health among the study persons was not explored [14]. Poor oral health can cause frequent spontaneous bacteremias during daily activities (chewing, brushing) that may cumulatively pose a greater risk for IE than occasional dental procedures [6].

This study aimed to determine whether poor oral health—specifically periodontal disease and caries - is associated with VGS-IE among high-risk individuals in Sweden. Nationwide health and dental registers were linked to conduct a case-control study, nested in a cohort study decribed previously [3].

## Methods

### Ethical approval

The study was approved by the Regional Ethical Review Board in Stockholm (DNR 2818/370-31). Informed consent was waived in accordance with board guidance.

### Study design

This study was a nationwide, nested case-control analysis conducted within a cohort of individuals at high risk of IE, using Swedish national health and dental registers. The study adhered to the Strengthening the Reporting of Observational Studies in Epidemiology (STROBE) guidelines for reporting observational studies.

### Study population

The study population has been described in detail previously [3]. In brief, the study included all adults residing in Sweden on July 1, 2008, who had one or more of the following high-risk conditions: prior IE, prosthetic heart valve implantation, or CHD, as recorded in the National Patient Register (NPR) or the Medical Birth Register (MBR) (see Table 1 for codes). Participants were followed through December 31, 2018.

**Table 1.** ICD- and surgical procedural codes used to identify individuals at high risk of IE.

**Endocarditis—ICD-10 codes**
I33.0, I33.9, I38.9
**Cyanotic congenital heart disease—ICD-10 codes**
Q20, Q20.1, Q20.2, Q20.4, Q24, Q21.2, Q13, Q21.3, Q21.8, Q26.2
**Prosthetic heart valve—ICD-10 codes**
Z95.2, Z95.3, Z95.4
**Prosthetic heart valve—surgical procedure codes**
FAA10, FCA60, FCA70, FCC70, FC76,
FCD00, FDC10, FGA96, FGE10, FGE120,
FGE96, FHB80, FHF00, FJF00, FJF10,
FJF12, FJF20, FJF96, FKD000, FKD020,
FKD96, FMD00, FMD12, FMD13, FMD20,
FMD33, FMD33, FMD40, FMD96, FMD96

Codes for the inclusion of high-risk individuals. Corresponding codes for earlier episodes of disease classified according to ICD-7-9 were identified using translator tables produced by the National Board of Health and Welfare. (ICD = international classification of diseases).

The final cohort comprised 64,634 individuals. Individuals <18 years were not included in the study, as paediatric cases are not consistently captured in the Swedish Registry of Infective Endocarditis (SRIE). Individuals who underwent IDPs within three months prior to IE diagnosis (*n* = 11) were excluded to minimise reverse causation bias. In addition, individuals aged ≤23 years (*n* = 10,871) were excluded, because the Swedish Dental Health Register (DHR) does not capture dental treatments in younger individuals receiving free dental care.

Participants were censored at the earliest of death, emigration, or end of the study period.

### Exposure assessment

Poor oral health was defined using a validated treatment-based method [15]. Utilising the DHR, study persons were classified as having severe oral disease if they had ≥2 treatment episodes for caries or periodontal disease, including ≥1 tooth extraction coded with a diagnosis of caries or periodontitis (Table 2). This definition was chosen to identify individuals with disease severe enough to result in tooth loss. The definition was validated against clinical data in 2025, for periodontitis but not for caries [15]. The positive predictive value for severe periodontitis was 84% when using Stage III–IV as gold standard, and 96% compared for Stage II–IV. The negative predictive value was 63% for Stage II–IV and 86% for Stage III–IV [15].

**Table 2.** Dental codes used to classify severe periodontitis, severe caries, and dental examination.

Condition	Classification criteria	Codes
Severe periodontitis	≥1 dental surgical procedure with periodontitis diagnosis, and ≥2 extensive or very extensive non-surgical periodontal treatments	Procedure codes: 342, 343 (scaling); 400–499 (oral surgery). Diagnosis code: 3043 (periodontitis)
Severe caries	≥1 dental surgical procedure with caries diagnosis, and ≥2 treatments with caries diagnosis	Procedure codes: >0 (dental examination/treatment); 400–499 (oral surgery). Diagnosis codes: 4001, 4002, 4011, 4012, 4022 (caries)
Dental examination	At least one examination procedure	Procedure codes: 101 (dental examination)

Severe periodontitis was defined as ≥1 dental surgical procedure with a periodontitis diagnosis combined with ≥2 extensive or very extensive non-surgical periodontal treatments. Severe caries was defined as ≥1 dental surgical procedure with a caries diagnosis combined with ≥2 additional treatments with a caries diagnosis. Dental examination was defined as the presence of at least one recorded dental examination procedure. The specific procedure and diagnosis codes used for classification are listed.

### Outcome definition

The primary outcome was first hospitalisation for IE during 2008–2018, as recorded in the SRIE. An additional analysis was conducted on VGS- IE. To avoid misclassification of procedure-related IE, a 3-month grace period was applied after high-risk cardiac surgery or prior IE hospitalisation [16].

### Case-control sampling

Nested case-control sampling was performed using incidence density sampling. For each incident IE case during follow-up, up to 10 controls were randomly selected from the risk set, matched on age, sex, and cardiac risk group. Controls were eligible to later become cases. The date of the IE admission served as the index date for each case and matched controls. Exposure status was assessed until three months prior to the index date.

### Covariates

The multivariable models adjusted for the following comorbidities; pacemaker, diabetes, history of heart disease, rheumatic fever, substance abuse, intravenous catheter, heart transplant as registered in the MBR or NPR since 1964. To minimise residual confounding, models also ajusted for age, sex, and highest educational attainment (categorised as primary, secondary, tertiary), obtained from Statistics Sweden.

### Statistical analysis

Conditional logistic regression was used to estimate odds ratios (ORs) and 95% confidence intervals (CIs) for the association between poor oral health and IE.

Complete case analysis was performed; all individuals included in the analysis had complete data on all relevant variables. No imputation of missing data was required.

The statistical analysis was carried out using Stata/IC 15 (StataCorp. 2017. Stata Statistical Software: Release 15. College Station, TX: StataCorp LLC).

### Sensitivity analyses

Sensitivity analyses included: (1) restricting to individuals with ≥1 dental examination recorded prior to cohort entry (to ensure oral health data completeness); (2) restricting to definite IE cases according to SRIE criteria; (3) stratified analyses by risk group.

A stratified ad hoc analysis was conducted by assessing the association between oral health and recurrent IE within the group with previous IE. This group contained individuals prothetic heart valves, as well as CHD. The analyses were stratified by presence of these comorbidities to examine whether the effect of poor oral health on IE recurrence varied across risk profiles.

### Data sources and linkage

Data were obtained from the following nationwide registers: the NPR (high-risk conditions), MBR (CHD), SRIE (outcomes), Total Population Register (demographics), and DHR (oral health exposure). Linkage was performed using unique national registration numbers.

#### SRIE

The register was used to identify cases of VGS-IE among the individuals at high risk. The Swedish Society for Infectious Diseases established the SRIE in 1995. All 30 infectious disease departments in Sweden have participated in the registry since it was created and the coverage has been estimated to 88% of all cases of IE [17]. These departments are responsible for providing care to patients with severe infections, and patients who require emergency surgery for IE are typically treated in these departments before and/or after the surgery. All cases are documented using standardised forms at the time of discharge and again after follow-up (average of 3 months after treatment). The form includes information about risk factors, the presence of prosthetic valves and other implantable cardiac devices, and the type of prosthetic valve. The microbial profile of the infection is determined using methods such as blood cultures, cultures from valves during surgery, and 16S RNA sequencing of tissue samples from valves.

Microbiological data were derived from blood-culture results recorded in the Swedish Endocarditis Registry and are primarily reported at the level of bacterial groups rather than individual species. Major categories included *Staphylococcus aureus* (methicillin-susceptible and methicillin-resistant), coagulase-negative staphylococci, alpha-haemolytic streptococci, enterococci, HACEK organisms, and other Gram-positive and Gram-negative bacteria.

Alpha-haemolytic streptococci represent a heterogeneous group that largely corresponds to viridans streptococci, which are commonly part of the oral flora, but may also include other alpha-haemolytic streptococci that are difficult to distinguish at species level in routine clinical practice. In the present study, *Streptococcus bovis* (i.e. *S. gallolyticus* group) and *Streptococcus pneumoniae* were recorded as separate categories and were therefore excluded from analyses intended to reflect oral streptococci. Streptococcus-like genera such as *Granulicatella* and *Abiotrophia* were also recorded separately and separated from the oral streptococci subgroup.

#### DHR

Data on dental procedures and diagnoses was obtained from the Swedish DHR.

The DHR is a national database containing information on dental care services compensated by the Swedish state. Children and adults under the age of 24 receive free dental care publicly funded through taxes, not included in the DHR. The DHR was established in 2008 and is administered by the Social Insurance Agency (SIA). All dental clinics are required to report procedural codes (Table 2) to the SIA to receive financial compensation from the national dental care subsidy, that covers all Swedish residents insured and covered by the Swedish social insurance system. The coverage is estimated to be nearly complete nationally [18]. Electronic dental record systems are connected to the SIA, and procedural data is transferred automatically prior to debit. The data is then transferred monthly from the SIA to the DHR held by the National Board of Health and Welfare, responsible for maintaining the national health data registers. The DHR is governed by legislation issued by the Swedish government and is used for a variety of purposes, including the evaluation and planning of health care services, monitoring public health, and conducting research. Electronic reporting is mandatory for dental health professions [18].

#### Total population register (TPR)

Data on sex, age, educational attainment, and date of death or emigration was obtained from the TPR held by Statistics Sweden, the government agency responsible for developing, producing, and disseminating official statistics and other government statistics in Sweden.

#### NPR/MBR

The NPR, which covers >99% of inpatient discharges in Sweden, was used to identify the risk groups, as well as comorbidities (heart disease, pacemaker, rheumatic fever, diabetes, substance use, catheter, and heart transplant) adjusted for in the analysis. The Swedish MBR, covering prenatal and neonatal health since 1973, was queried to capture CHD not recorded in adulthood (see Table 1 for codes). Both registers are held by the National Board of Health and Welfare.

### Results

#### Cohort characteristics

Between the 1^st^ of and the 1^st^ of , 220 cases of VGS-IE occurred among the 64,634 individuals at high risk. At baseline, 61% of the cohort were male, and the median age was 67 years (interquartile range 58–79). The high-risk cohort included approximately 68% with prosthetic heart valves, 13% with congenital heart disease, and 19% with a history of prior endocarditis. Baseline oral health status varied by risk group (Table 3). Overall, 7% of the cohort met the definition of severe oral disease. The prevalence of VGS-IE and all-cause IE among the risk groups is displayed in Table 4.

**Table 3.** Characteristics of the study participants.

Characteristic	CHD No IE	CHD IE	Prosthetic valve No IE	Prosthetic valve IE	Previous IE No IE	Previous IE IE
N	120	12	5,219	527	3,340	342
Age, median (IQR)	41.13 (31.75–52.29)	33.83 (31.08–45.13)	71.16 (62.50–77.75)	70.83 (62.58–77.52)	58.48 (44.75–72.51)	58.35 (42.75–71.68)
Male	100 (83.3)	10 (83.3)	3,802 (72.8)	384 (72.9)	2,325 (69.6)	238 (69.6)
Female	20 (16.7)	2 (16.7)	1,417 (27.2)	143 (27.1)	1,015 (30.4)	104 (30.4)
**Educational attainment**						
Missing	7 (5.8)	1 (8.3)	47 (0.9)	9 (1.7)	60 (1.8)	5 (1.5)
Primary/secondary	29 (24.2)	3 (25.0)	2,150 (41.2)	183 (34.7)	1,117 (33.4)	141 (41.2)
Upper secondary	54 (45.0)	6 (50.0)	1,970 (37.7)	230 (43.6)	1,435 (43.0)	145 (42.4)
Post- secondary	30 (25.0)	2 (16.7)	1,052 (20.2)	105 (19.9)	728 (21.8)	51 (14.9)
Caries only	0 (0.0)	0 (0.0)	125 (2.4)	14 (2.7)	92 (2.8)	25 (7.3)
Periodontitis only	0 (0.0)	0 (0.0)	51 (1.0)	8 (1.5)	23 (0.7)	7 (2.0)
Both caries & periodontitis	0 (0.0)	0 (0.0)	4 (0.1)	0 (0.0)	3 (0.1)	2 (0.6)
CHD (lower risk)	115 (95.8)	12 (100.0)	203 (3.9)	20 (3.8)	304 (9.1)	24 (7.0)
Pacemaker	12 (10.0)	1 (8.3)	1,396 (26.7)	180 (34.2)	587 (17.6)	108 (31.6)
Rheumatic fever	3 (2.5)	1 (8.3)	22 (0.4)	2 (0.4)	42 (1.3)	5 (1.5)
Diabetes	4 (3.3)	1 (8.3)	900 (17.2)	117 (22.2)	372 (11.1)	67 (19.6)
Substance abuse	2 (1.7)	0 (0.0)	79 (1.5)	14 (2.7)	124 (3.7)	35 (10.2)
IV catheter/ haemodialysis	1 (0.8)	0 (0.0)	33 (0.6)	25 (4.7)	66 (2.0)	28 (8.2)
Heart transplant	2 (1.7)	0 (0.0)	73 (1.4)	5 (0.9)	41 (1.2)	3 (0.9)

**Table 4.** Distribution of IE and VGS-IE among individuals by oral health.

Cardiac risk group	Outcome	Cases oral disease n/N (%)	Non-cases oral disease n/N (%)	Crude OR (95% CI)	*p*
Congenital heart disease	All-cause IE	0/12 (0.00%)	0/120 (0.00%)	NE	–
Congenital heart disease	VGS-IE	0/5 (0.00%)	0/50 (0.00%)	NE	–
Prosthetic valve	All-cause IE	22/527 (4.17%)	168/5,270 (3.19%)	1.32 (0.84–2.08)	0.23
Prosthetic valve	VGS-IE	6/138 (4.35%)	76/1,380 (5.51%)	0.78 (0.33–1.83)	0.57
Previous endocarditis	All-cause IE	34/342 (9.94%)	102/3,420 (2.98%)	3.59 (2.68–4.80)	<0.001
Previous endocarditis	VGS-IE	10/77 (12.99%)	48/768 (6.25%)	2.24 (1.08–4.63)	0.03

Summary of all-cause infective endocarditis (IE) and *Viridans* group streptococcal IE (VGS-IE) incidence by cardiac risk group and oral health status. The table displays the percentage of individuals with all-cause IE and VGS-IE within each cardiac risk group, stratified by oral health status (presence or absence of severe oral disease). Oral disease was defined based on severe periodontitis and/or severe caries. Percentages reflect the proportion of affected individuals within each subgroup. NE; not estimable.

### Association of oral health with IE

**Figure 1.**
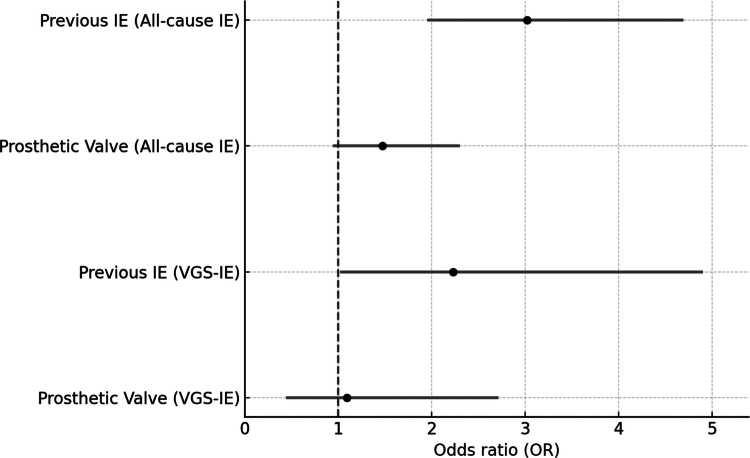
Association between poor oral health and risk of infective endocarditis (IE), by risk group and outcome. Forest plot of odds ratios (ORs) and 95% confidence intervals (CIs) from matched conditional logistic regression models. Separate estimates are shown for all-cause infective endocarditis (IE) and *Viridans* group streptococcal IE (VGS-IE), by risk group (prosthetic valve, previous IE). Congenital heart disease is not displayed as the association was not estimable due to collinearity. The vertical dashed line indicates OR = 1 (no association).

The adjusted results of the logistic regression analyses are found in Figure 1. Analysis of the association between poor oral health and IE resulted in consistent patterns across VGS-IE and all-cause IE results. Among individuals with prior IE, poor oral health was associated with both all-cause IE and VGS-IE. All-cause IE: adjusted OR 3.02 (95% CI 1.95–4.69). VGS-IE: adjusted OR 2.23 (95% CI 1.02–4.90). Among those with prosthetic valves, no significant associations were observed (all-cause IE: adjusted OR 1.47 [95% CI 0.94–2.30]; VGS-IE: adjusted OR 1.09 [95% CI 0.44–2.71]). In the CHD group, no estimable association could be obtained due to sparse data.

Over the follow-up period (median follow-up 5.4 years), a total of 881 first-time IE episodes occurred in the cohort. IE incidence was highest among those with a past history of endocarditis, whereas the rest were first-ever IE events in those with prosthetic valves or CHD but no previous IE.

Overall, the microbiological profile of IE cases showed that approximately 26% were caused by *Viridans* group streptococci. Cases in the poor oral health groups had a higher proportion of VGS-IE (29% compared to 25%), although this difference was not statistically significant (*p* = 0.49).

Table 4 illustrates the cumulative incidence of IE over time in those with poor oral health versus those without, stratified by high-risk subgroup. In individuals with a history of prior IE, poor oral health was associated with a markedly higher risk of recurrent endocarditis. By five years of follow-up, approximately 10% of patients with both prior IE and poor oral health had experienced an IE recurrence, compared to about 3% of those with prior IE but without severe oral disease (Table 4).

### Sensitivity analyses

The results were robust across multiple sensitivity analyses. When the cohort was restricted to individuals who had at least one documented dental examination prior to study entry (to ensure complete oral health data capture), the associations between poor oral health and recurrent IE remained statistically significant (*p* < 0.001—*p* = 0.05). Additionally, when using only definite IE cases confirmed in the SRIE (excluding IE diagnoses not meeting Duke criteria), the interpretation and direction of the results were unchanged.

### Stratification among those with previous IE

Among individuals with a history of IE, recurrence differed by co-existing cardiac conditions. Using four mutually exclusive strata - previous IE only (no CHD/valve), previous IE + congenital heart disease (CHD), previous IE + prosthetic valve, and previous IE + CHD + prosthetic valve - the all-cause recurrent IE risk was 4.0% (427/10,624) overall and varied as follows: prosthetic valve 8.0% (338/4,244), CHD + prosthetic valve 3.1% (6/192), CHD only 2.0% (4/196), and previous IE only 1.3% (79/5, 992).

For VGS-IE, events were rarer - 0.91% (101/11, 102) overall - but showed a similar pattern (*p* < 0.001): prosthetic valve 1.78% (78/4,373), CHD only 1.52% (3/198), CHD + prosthetic valve 0.51% (1/197), and previous IE only 0.30% (19/6, 334).

### Stratification among those with prosthetic valves

Within the prosthetic-valve group, poor oral health was associated with all cause IE after adjustment for prior IE and covariates (OR 2.14, 95% CI 1.24–3.69). In analyses stratified by prior IE status, the association was evident among individuals with prior IE (OR 2.18, 95% CI 1.24–3.84), whereas estimates among those without prior IE were imprecise (OR 1.65, 95% CI 0.18–15.36). The interaction between poor oral health and prior IE was not statistically significant (*p* = 0.81).

## Discussion

This nationwide study provides evidence that poor oral health is associated with recurrent IE, while it does not appear to influence IE risk in individuals with prosthetic valves without previous IE. In an additional analysis restricted to individuals with prosthetic valves, poor oral health was associated with IE among those with prior IE, whereas no clear association was observed among those without prior IE; however, estimates in the latter subgroup were imprecise due to sparse events. Although the study contined almost 19.000 participants with CHD, it was underpowered to study the association in that risk group. The CHD group consisted of younger individuals (mean age 31 years), with few meeting the definition of severe oral disease and VGS-IE (0.1% and 0% respectively).

The results align with previous evidence on dental procedure-related bacteremia and subsequent IE. A recent Swedish nested case-crossover study examined the timing of IDPs before IE episodes in high-risk individuals and found no temporal association between undergoing a dental procedure and the onset of VGS-IE within the following months [14]. In that study, 4–5% of patients with oral streptococcal endocarditis had undergone an IDP in the preceding three months—a proportion nearly identical to that observed in matched controls without IE. As the vast majority appeared to arise sporadically, this highlights the likely importance of continuous everyday exposures (such as tooth brushing, chewing, and ongoing oral infections) in causing bacteremia that can lead to IE. Poor oral health, by increasing the bacterial load and frequency of spontaneous bacteremia, could raise the risk of IE recurrence in those with prior valve damage.

The current study has several strengths. By using comprehensive national registers with virtually complete coverage of hospital admissions and dental treatments, selection and information biases were minimised. The large sample of high-risk patients and extended follow-up provided power to detect associations and allowed sensitivity analyses.

Furthermore, the outcome definition was robust, capturing IE cases from the specialised endocarditis quality register, and applying a grace period after high-risk surgery to avoid misclassification. Excluding individuals without pre-cohort dental records did not materially change the effect estimates, alleviating concern that the findings were driven by differential healthcare utilisation. In addition, the use of incidence density sampling and complete case analysis ensured that follow-up was nearly complete, with minimal risk of selection bias due to loss to follow-up.

The study has limitations. First, although validated against clinical data, the definition of poor oral health was based on treatment proxies (frequency of caries and periodontal interventions) rather than direct clinical oral examinations. Second, it is possible that some individuals with poor oral health did not seek dental care and were therefore misclassified; however, such misclassification would bias the association toward the null. This concern was addressed by conducting sensitivity analyses restricted to individuals with prior dental examination. Third, the definitions of periodontitis and caries were strict, capturing only individuals with extensive treatment and thus likely severe disease. Broader definitions may identify more cases but at the cost of lower specificity and sensitivity. Notably, while the definition of severe periodontitis has been validated against clinical data, the definition of caries has not been validated. Third, the registers lacked data on smoking, a known risk factor for periodontitis and a potential confounder of the association between poor oral health and IE. If smoking independently increases IE risk, residual confounding could partially explain the observed associations. However, smoking prevalence in Sweden is low; approximately 6% of adults are daily smokers and 2–4% are occasional smokers. Additionally, matching on age, sex, and educational attainment likely mitigated some of the potential confounding by smoking. Fourth, in the SRIE, species-level identification was not consistently available throughout the study period, and information on laboratory identification methods (e.g. pre- versus post–matrix-assisted laser desorption/ionisation time-of-flight mass spectrometry [MALDI-TOF MS]) was not captured in the registry. Accordingly, analyses were based on the reported group-level classifications, and potential species-level misclassification was considered a limitation.

The generalisability of our findings is strengthened by the use of nationwide registers with high coverage and validated data sources. Although a significant association was found only in the risk group with previous IE, stratification revealed that 40% of the individuals in this group also had a prothethic heart valve. The study population included vitually all individuals in Sweden with key cardiac risk factors for IE, and the Swedish healthcare context provides subsidised access to both dental and medical care, minimising selection bias. However, generalisability to countries with differing healthcare systems, or smoking prevalence may be limited. Furthermore, the internal validity for individuals with CHD was lacking in this study. There are a few reasons for the finding of few cases of IE and poor oral health in the CHD group. First, the CHD sample mean age at inclusion was 41 years (prosthetic heart valve group: 69 years, prior IE group: 58 years). Both poor oral health and IE are less frequent among younger individuals. It is also likely that in this group, growing up with a known complex congenital heart disease, awareness of the importance of oral health, and adherence to recommendations for maintaining it, prevented many from having poor enough oral health to meet the strict criteria.

## Conclusion

In conclusion, among individuals with previous IE, poor oral health was associated with an increased risk of IE recurrence, whereas no association was confirmed among patients with prosthetic valves and no previous IE. Promoting good oral health may be an important component of secondary prevention in patients with a history of IE. These findings provide epidemiological support for continuing to integrate oral health considerations into preventive care for cardiac patients.

## Data Availability

The study was conducted using national Swedish registries. The data that support the findings of this study are available from the SRIE, Statistics Sweden and the Swedish National Board of Health and Welfare but restrictions apply to the availability of these data, which were used under license for the current study, and so are not publicly available.

## Licence for publication

The Corresponding Author has the right to grant on behalf of all authors and does grant on behalf of all authors, a world-wide licence to the Publishers and its licencees in perpetuity, in all forms, formats and media (whether known now or created in the future), to i) publish, reproduce, distribute, display and store the Contribution, ii) translate the Contribution into other languages, create adaptations, reprints, include within collections and create summaries, extracts and/or, abstracts of the Contribution, iii) create any other derivative work(s) based on the Contribution, iv) to exploit all subsidiary rights in the Contribution, v) the inclusion of electronic links from the Contribution to third party material where—ever it may be located; and, vi) licence any third party to do any or all of the above.
